# Worming forward: amyotrophic lateral sclerosis toxicity mechanisms and genetic interactions in *Caenorhabditis elegans*

**DOI:** 10.3389/fgene.2014.00085

**Published:** 2014-04-17

**Authors:** Martine Therrien, J. Alex Parker

**Affiliations:** ^1^Départment de Pathologie et Biologie Cellulaire, CRCHUM-Centre Hospitalier de l'Université de Montréal, Université de MontréalMontréal, QC, Canada; ^2^Départment de Pathologie et Biologie Cellulaire, Départment de Neurosciences, CRCHUM-Centre Hospitalier de l'Université de Montréal, Université de MontréalMontréal, QC, Canada

**Keywords:** *C. elegans*, ALS (Amyotrophic lateral sclerosis), TDP-43, FUS, C9orf72, SOD1, genetic networks, motor neuron disease

## Abstract

Neurodegenerative diseases share pathogenic mechanisms at the cellular level including protein misfolding, excitotoxicity and altered RNA homeostasis among others. Recent advances have shown that the genetic causes underlying these pathologies overlap, hinting at the existence of a genetic network for neurodegeneration. This is perhaps best illustrated by the recent discoveries of causative mutations for amyotrophic lateral sclerosis (ALS) and frontotemporal degeneration (FTD). Once thought to be distinct entities, it is now recognized that these diseases exist along a genetic spectrum. With this wealth of discoveries comes the need to develop new genetic models of ALS and FTD to investigate not only pathogenic mechanisms linked to causative mutations, but to uncover potential genetic interactions that may point to new therapeutic targets. Given the conservation of many disease genes across evolution, *Caenorhabditis elegans* is an ideal system to investigate genetic interactions amongst these genes. Here we review the use of *C. elegans* to model ALS and investigate a putative genetic network for ALS/FTD that may extend to other neurological disorders.

## Introduction

Amyotrophic lateral sclerosis (ALS) is a neurodegenerative disorder affecting 1–2/100,000 individuals. Most cases of ALS are sporadic, but 10% of cases are familial (Turner et al., 2013b). Mutations in the gene *superoxide dismutase 1 (SOD1)* were identified in 1993 (Rosen et al., 1993) as the first cause of familial ALS. Thanks to the recent advances in genetics, more than 20 genes are now linked to ALS (Chen et al., 2013) (Table 1). Genes recently shown to be mutated in ALS include the DNA/RNA binding proteins *TAR DNA binding protein 43 (TARDBP)* and *Fused-in-sarcoma (FUS)* (Kabashi et al., 2008; Sreedharan et al., 2008; Vance et al., 2009). More recently, mutations in *C9ORF72* have turned out to be a major cause of familial and sporadic ALS (DeJesus-Hernandez et al., 2011; Renton et al., 2011).

**Table 1:** **ALS genes and their *C. elegans* orthologs**.

**Human gene**	**Function**	***C. elegans* gene**
**MOST COMMON ALS GENES**		
*SOD1*	Superoxide metabolism	*sod-1*
*TARDBP*	RNA metabolism	*tdp-1*
*FUS*	RNA metabolism	*fust-1*
*OPTN*	Vesicular transport	*–*
*VCP*	Vesicular transport	*cdc-48.1/2*
*UBQLN2*	Proteasome	*ubqnl-1*
*C9ORF72*	Unknown, DENN protein	*alfa-1*
*SQSTM1*	Autophagy	*sqst-2*
*PFN1*	Cytoskeleton dynamics	*pfn-1*
**OTHER GENES INVOLVED IN ALS**		
*DCTN1*	Cytoskeleton dynamics	*dnc-1*
*ALS2*	Endocytosi*s*	*–*
*CHMP2B*	Vesicular transport	*–*
*FIG4*	Vesicular transport	*C34B7.2*
*HNRNPA2B1*	RNA metabolism	*hrp-1*
*ELP3*		*–*
*SETX*	RNA processing	*–*
*HNRNPA1*	RNA processing	*hrp-1*
*ATXN2*		*atx-2*
*ANG*	Blood vessels formation	*–*
*SPG11*	DNA damage	*–*
*VAPB*	Vesicular transport	*vpr-1*
*NEFH*	Cytoskeleton dynamics	*H39E23.3*
*ARHGEF28*	RNA metabolism	*rhgf-1*

ALS is characterized by the selective loss of motor neurons in the motor cortex, the brainstem and the spinal cord, the loss of myelin in the spinal cord, and the presence of neuroinflammation (Robberecht and Philips, 2013). Onset of the disease usually begins in the lower limb and spreads toward the upper motor neurons leading to muscle weakness, fasciculation, and wasting. Death occurs 3–5 years after the beginning of the symptoms (Kiernan et al., 2011) and is caused by respiratory failure due to denervation of the respiratory muscles.

50% of ALS patients show cognitive impairment, of which 15% met the criteria of frontotemporal dementia (FTD) (Ringholz et al., 2005). FTD is a group of non-Alzheimer dementias characterized by atrophy of the frontal and/or temporal lobes causing mid-life behavioral changes or language impairment (Warren et al., 2013). Over the past few years, the identification of *TDP-43*, *C9ORF72* and *UBQLN2* as genes causing ALS and FTD has suggested a similarity for both diseases (Morris et al., 2012). Similar pathogenic mechanisms have been suggested for ALS and FTD (Van Langenhove et al., 2012; Ling et al., 2013) but so far it is unclear how patients with the same genetic mutations can have either ALS, FTD or both.

The genes involved in ALS have diverse functions and we still do not know how they interact to cause motor neuron degeneration. Most of the research over the past 20 years has focused on the toxicity caused by mutant SOD1. Among the proposed mechanisms of toxicity are mitochondrial dysfunction, axonal dysfunction, excitotoxicity and neuroinflammation (Turner et al., 2013b). However, TDP-43, FUS, and C9ORF72 proteins seem to point toward RNA toxicity (Ling et al., 2013). Most importantly, only one drug, riluzole, is used to slow disease progression and has only modest effects (Kiernan et al., 2011). Diagnosis is difficult and requires an experienced neurologist to differentiate between ALS and other neurological diseases (Turner et al., 2013a). It is thought that the clinical manifestations of ALS are downstream events that occur much later after the initial insult to the nervous system (Turner et al., 2013a). Therefore, the identification of biomarkers is essential for the rapid, early diagnosis of ALS, and the identification of new drugs limiting the degeneration of motor neuron is an essential unmet need for ALS patients.

To understand better the impact of the genetic mutations on the function of the different proteins involved in ALS, *in vivo* models have proved to be essential. Ever since the first SOD1 mouse was developed in 1994 (Gurney et al., 1994), several groups have tried to investigate ALS pathogenesis by expressing different ALS related mutations in mice, an approach that has recently been extended to other genes including TDP-43 and FUS for example. While the over expression of wild type SOD1 causes a mild denervation of neurons (Epstein et al., 1987), the over expression of SOD1^G93A^ causes a loss of motor neurons, neuroinflammation, and reduces life span (Gurney et al., 1994; Guo et al., 2009). One model expressing mutant TDP-43Q^331K or M337V^ in the mouse central nervous system has shown selectivity for large caliber motor neuron neurodegeneration (Arnold et al., 2013), while others over expressing mutant TDP-43^G348C, A315T^ and FUS^R512C, 14Δ^ have limited neuronal loss (Swarup et al., 2011; Verbeeck et al., 2012). Some rodent models display relevant ALS pathology, but given the time and expense to develop models for many of the recently discovered ALS genes, not to mention the difficulty of manipulating several genes at once, some laboratories have turned to simpler organisms to model ALS toxicity.

One model showing increasing popularity is the nematode *Caenorhabditis elegans*. This 1 mm long worm has a painstakingly characterized, invariant cell lineage that includes 302 neurons. The nervous system, its interconnections and its synapses are well studied which makes it an ideal model to study mechanisms of neuronal toxicity. The *C. elegans* genome was the first to be fully sequenced in 1998 and includes more than 19,000 genes on 6 chromosomes (*C. elegans* Sequencing Consortium, 1998). Since then, deletion mutants have been produced for many *C. elegans* genes and approximately 80% of *C. elegans* genes have a human homolog (Lai et al., 2000) (Shaye and Greenwald, 2011). *C. elegans* behavior is well studied and many experimental assays are available, including for worm locomotion. Worms initiate movement by bending their body to advance forward in a sinusoidal pattern, a process that is orchestrated by GABAergic and cholinergic neurons. Cholinergic neurons initiate the contraction along the dorsal or ventral body wall muscles while the GABAergic neurons send an inhibitory signal on the opposite side (Jorgensen, 2005).

*C. elegans* has been an important tool for the characterization of many neurodegenerative disorders (Li and Le, 2013). Many protein-misfolding disorders have been modeled in worms including Alzheimer's disease, Parkinson's disease, Huntington's disease and different spinocerebellar ataxias. Also, the toxicity of non-coding mutations in *C. elegans* resemble the toxicity in mammalian tissues (Wang et al., 2011b). Since many cellular stress and survival pathways are conserved in worms, our group and others have used *C. elegans* to model ALS. This review aims to summarize the work done modeling ALS in *C. elegans* and highlights the future possibilities and applications.

## Use of *C. elegans* to model SOD1 toxicity

SOD1 is an enzyme that catalyzes the conversion of O_2_- into O_2_ and H_2_O_2_. More than 160 mutations causative for ALS have been found in *SOD1* since 1993 (Al-Chalabi et al., 2012). Phenotypic heterogeneity is observed among *SOD1* mutation carriers where *SOD1*^A4V^ seems to cause an aggressive form of ALS while *SOD1*^D90A^ causes a milder, long duration ALS (Renton et al., 2014). It is hypothesized that *SOD1* mutations cause toxicity through a gain of function, even though a loss of enzyme activity have been observed in patients and some models (Saccon et al., 2013). Many pathogenic mechanisms have been hypothesized but no consensus has been reached, although it is thought that the misfolding of mutant SOD1, and sometimes wild type SOD1, may be an important first step of the pathogenesis observed in patients (Pickles and Vande Velde, 2012). Based on pathological evidence, it is now acknowledged that ALS caused by mutations in SOD1 is a distinctive form of ALS (Mackenzie et al., 2007).

Several groups have used *C. elegans* to model SOD1 toxicity (Table 2) starting with Oeda and colleagues who showed that the ubiquitous expression of human mutant SOD1 impairs the worm's response to oxidative stress and causes protein aggregates (Oeda et al., 2001). It was later shown that expression of mutant SOD1 throughout the worm's entire nervous system resulted in locomotion defects and impaired neuronal transmission (Wang et al., 2009). Interestingly, the formation of aggregates seemed to be restricted to certain mechanosensory neurons despite the pan neuronal expression of SOD1. Other models are non-neuronal in nature and have relied on the expression of SOD1 proteins in the body wall muscles where it was observed that distinct SOD1 mutations have varying propensities to aggregate (Gidalevitz et al., 2009). More recently a *C. elegans* model was generated based on the expression of SOD1 in the worm's motor neurons showing neurodegeneration in the absence of caspases (Li et al., 2013a), an intriguing finding since the motor neuron loss observed in mouse models is associated with caspase activation (Pasinelli et al., 2000). Whether this reflects a difference between invertebrate and vertebrate systems, or reflects a novel mechanism of neurodegeneration remains to be determined.

**Table 2:** **Summary of transgenic SOD1 models**.

**Study**	**Promoter**	**Gene**	**Motor phenotype**	**Aggregation**	**Neuro-degeneration**	**Synaptic dysfunction**
Oeda et al., 2001	*hsp-16.2:* all tissues except the germline	SOD1^A4V^	n.d	n.d.	n.d	n.d
		SOD1^G37R^	n.d	n.d	n.d	n.d
		SOD1^G93R^	n.d	n.d	n.d	n.d
	*myo-3:* muscle cells	SOD1^A4V^	n.d	yes	n.a	n.a
Wang et al., 2009	*snb-1:* entire nervous system	SOD1^WT^	No	No	n.d	Normal
		SOD1^G83R^	Yes	Yes	n.d	Impaired
Gidalevitz et al., 2009	*unc-54:* muscle cells	SOD1^WT^	No	No	n.a.	n.a
		SOD1^G85R^	Yes	Yes	n.a.	n.a.
		SOD1^G93A^	Yes	Yes	n.a.	n.a.
		SOD1^127X^	Yes	Yes	n.a.	n.a.
Li et al., 2013a	*unc-25:* GABAergic motor neurons	SOD1^WT^	Yes	Yes	Yes	n.d.
		SOD1^G93A^	Yes	Yes	Yes	n.d.

*n.d., not determined; n.a., not applicable*.

The *C. elegans sod-1* gene has a similar function to human *SOD1*. *sod-1* loss of function mutants have increased O_2_- levels, shorter lifespan and are sensitive to some environmental stresses (Yanase et al., 2009). Inversely, overexpression of the worm *sod-1* increases lifespan and increases the level of H_2_0_2_, the by-product of the catalase reaction of SOD1. However, the increased lifespan seems to be independent of SOD-1 catalase activity, but may be due to altered endoplasmic reticulum (ER) stress signaling (Cabreiro et al., 2011). Interestingly, Van Raamsdonk et al. have generated a *sod* null worm, where all five *C. elegans sod* genes have been mutated and these worms have a normal lifespan and response to oxidative damage but are sensitive to many acute environmental stresses (Van Raamsdonk and Hekimi, 2012).

In summary, many aspects of SOD1 function and toxicity are conserved in worms, but some questions remain. It is known that mutant SOD1 is found in association with the mitochondria in SOD1 mouse model and ALS patients (Pickles and Vande Velde, 2012). To our knowledge, no group has yet investigated the effects of human mutant SOD1 in worm mitochondria. However, it was recently shown that a cleavage product of *vpr-1*, the ortholog of VAPB also involved in ALS, affects mitochondrial organization in muscle cells (Han et al., 2013). A similar analysis of the different SOD1 transgenic models would be interesting and could help identify pathways and drugs that act specifically on this important aspect of ALS pathogenesis.

## Use of *C. elegans* to model TDP-43 toxicity

TDP-43 is a protein encoded by the *TARDBP* gene on chromosome 1. The protein contains two RNA binding domains, a glycine rich domain and nuclear export and import signals. TDP-43 is similar to the members of the ribonucleoprotein family. TDP-43 was identified in 2006 as the main constituents of sporadic and familial ALS/FTD aggregates (Neumann et al., 2006). In patients, the ubiquitinated aggregates are present in the most affected regions of the brain and spinal cord. These aggregates contain a hyperphosphorylated form of TDP-43 and the C terminus cleaved fragment (Neumann et al., 2006). In 2008, mutations in the *TARDBP* gene were linked to familial and sporadic ALS/FTD cases (Kabashi et al., 2008; Sreedharan et al., 2008; Sreedharan and Brown, 2013). So far, more than 40 mutations in *TARDBP* have been linked to ALS/FTD and most of them are found in the C terminus region of the protein, a region involved in protein-protein interactions (Al-Chalabi et al., 2012).

Under normal cellular conditions, TDP-43 protein shuttles from the nucleus to the cytoplasm. The normal function of TDP-43 is still unclear but the protein participates in transcription, miRNA processing, mRNA splicing, RNA transport and stress granule formation (Ling et al., 2013). The pathogenic effect of the mutant proteins is not well understood and it is still unclear if the toxicity is a gain of function, a loss of function, or both (Ling et al., 2013; Vanden Broeck et al., 2014). An important aspect of TDP-43 toxicity was discovered when characterizing TDP-43 wild type mice. Mice with elevated expression of wild type TDP-43 also have characteristics of TDP-43 mutant proteins (Xu et al., 2010). Therefore, expression level is important and should be considered when generating different transgenic models.

To clarify the toxicity caused by the expression of mutant TDP-43, several groups have developed *C. elegans* models (Table 3). In 2010, Ash and colleagues developed the first TDP-43 overexpression model in *C. elegans*. The pan neuronal expression of human TDP-43 and *C. elegans* TDP-1 resulted in worms with uncoordinated, slow movements and defasciculation of the GABAergic motor neurons (Ash et al., 2010). The results regarding the expression of human TDP-43 were confirmed by Liachko and colleagues who also observed motility defects and degeneration phenotypes from the expression of mutant TDP-43 proteins throughout the worms nervous system (Liachko et al., 2010). These phenotypes also highly correlated with protein phosphorylation levels where hyperphosphorylation increased the toxicity of mutant TDP-43 proteins similarly to what is observed in ALS patients (Liachko et al., 2010). The TDP-43 C terminus fragment shows another similarity with patients. Zhang and colleagues showed that the pan neuronal expression of human TDP-43 C′ fragment caused a phenotype similar to the expression of wild type or mutant TDP-43 (Zhang et al., 2011). Even though no neuronal loss was observed in the latter model, all strains displayed synaptic transmission abnormalities. In worms, GABAergic neurons seem to be particularly sensitive to the expression of TDP-43 (Liachko et al., 2010). To evaluate if the effect of TDP-43 expression in these neurons could cause locomotor defects, our group developed models in which human wild type or mutant TDP-43 were expressed specifically in GABAergic motor neurons as directed by an *unc-47* promoter (McIntire et al., 1997). Interestingly, the overexpression of mutant TDP-43, but not wild type TDP-43, caused an adult-onset, progressive paralysis phenotype accompanied by GABAergic neurodegeneration and synaptic transmission impairment (Vaccaro et al., 2012b). Finally, some of these models showed aggravation of the phenotypes during aging recapitulating an important feature of ALS and neurodegeneration (Liachko et al., 2010; Vaccaro et al., 2012b).

**Table 3:** **Summary of transgenic TDP-43 models**.

**Study**	**Promoter**	**Gene**	**Motor phenotype**	**Aggregation**	**Neuro-degeneration**	**Synaptic dysfunction**
Ash et al., 2010	*snb-1:* entire nervous system	TDP-1	Yes	n.d.	n.d.	n.d.
		TDP-43^WT^	Yes	n.d.	GABAergic	n.d.
		TDP-43^Δ^RRM1	No	n.d.	n.d.	n.d.
		TDP-43^Δ^RRM2	No	n.d.	n.d.	n.d.
		TDP-43^Δ^C terminus	No	n.d.	n.d.	n.d.
		TDP-43^no caspase^	Yes	n.d.	n.d.	n.d.
		TDP-43^no NLS^	No	n.d.	n.d.	n.d.
Liachko et al., 2010	*snb-1:* entire nervous system	TDP-43^WT^	Yes	Yes	No	n.d.
		TDP-43^G290A^	Yes	Yes	GABAergic and dopaminergic	n.d.
		TDP-43^A315T^	Yes	Yes	GABAergic and dopaminergic	n.d.
		TDP-43^M337V^	Yes	Yes	GABAergic and dopaminergic	n.d.
Zhang et al., 2012b	*snb-1:* entire nervous system	TDP-43^WT^	Yes	Yes	No	Impaired
		TDP-43^G331K^	Yes	n.d.	No	Impaired
		TDP-43^M337V^	Yes	n.d.	No	Impaired
		TDP-43^C terminus^	Yes	Yes	No	Impaired
Vaccaro et al., 2012c	*unc-47*: GABAergic neurons	TDP-43^WT^	No	No	No	No
		TDP-43^A315T^	Yes	Yes	GABAergic	Impaired

*n.d., not determined; NLS, nuclear localization signal; ΔRRM, deletion of RNA recognition motif; ΔC terminus, deletion of C terminus; no caspase, mutations in TDP-43 that block caspase cleavage*.

It is still unclear if the toxicity of mutant TDP-43 proteins in ALS patients arises from a gain of function, a loss of function or if both mechanisms are employed. The transgenic *C. elegans* models of TDP-43 are based on the overexpression of TDP-43 in worms and likely represent a gain of function rather than a loss of function. However, the *C. elegans* genome has an ortholog of TDP-43 called TDP-1. TDP-1 is a primarily nuclear protein expressed in most tissues including body wall muscles, pharynx and neurons (Vaccaro et al., 2012c; Zhang et al., 2012b). TDP-1 contains two RNA binding motifs, a nuclear localization signal and an export signal but lacks the glycine rich domain found in human TDP-43. TDP-1 seems to be functionally conserved because the expression of human TDP-43 can rescue the toxicity of a loss of function of a *tdp-1* mutant (Zhang et al., 2012b).

Mutant *tdp-1* animals show numerous phenotypes including slow development, and locomotion defects (Liachko et al., 2010; Zhang et al., 2012b). TDP-1 was also shown to be involved in lifespan and the cellular stress response. Somewhat paradoxically, worms lacking *tdp-1* have a longer lifespan but are more sensitive to oxidative and osmotic stresses (Vaccaro et al., 2012c; Zhang et al., 2012b). The expression of *tdp-1* can be induced by oxidative stress, either chemically or from activation of the ER stress response, and it is thought that chronic induction of *tdp-1* by stress is ultimately cytotoxic and reduces the worms lifespan (Vaccaro et al., 2012c). Furthermore, several studies have shown that wild type TDP-1 protein may contribute to the neurodegeneration elicited by mutant protein in *C. elegans*. Neurodegeneration was suppressed by deleting *tdp-1* from worms in several ALS models (Vaccaro et al., 2012c; Zhang et al., 2012b) as well as in a *C. elegans* model of Huntington's disease (Tauffenberger et al., 2013a) suggesting there may be genetic interactions amongst genes linked to neurodegeneration. Interestingly, a transcriptome analysis of *tdp-1(ok803)* showed that one of the biological process that was highly affected in the mutant worms was the ER unfolded protein response (Zhang et al., 2012b). ER stress and proteostasis have been a recurrent theme in ALS research (Matus et al., 2013; Musarò, 2013) which is of interest since sporadic and familial cases of ALS are known to have an abnormal ER stress response (Ilieva et al., 2007; Atkin et al., 2008; Hetz et al., 2009; Ito et al., 2009).

## Use of *C. elegans* to model FUS toxicity

After the identification of TDP-43, several groups examined related RNA-binding proteins for their potential contributions to ALS. In 2009, a protein with a similar function, FUS, was identified as causative of ALS (Kwiatkowski et al., 2009; Vance et al., 2009). Similar to TDP-43, FUS contains a RNA binding domain and a glycine rich domain but also has a two arginine glycine rich regions and one large glutamine, glycine, serine, tyrosine domain in N terminus. Because of their high degree of structural similarity, it was hypothesized that FUS and TDP-43 share common functions. It is known that FUS can bind DNA and RNA and is involved in many of the same RNA processing activities of TDP-43 (Ling et al., 2013). FUS transgenic models are relatively recent additions to the research field and much remains to be learned about the function of FUS and the implication of the mutant protein in neurodegeneration.

Two transgenic models have been developed in *C. elegans* for FUS (Table 4). Murakami et al. (2012) expressed several *FUS* mutations and two truncated FUS proteins throughout the worm's nervous system. Interestingly, only the mutations that caused aggregation resulted in motor phenotypes in worms. The motor phenotype could not be rescued by the expression of wild type *FUS* suggesting a gain of function mechanism. Our group confirmed a similar toxicity mechanism in models expressing *FUS* in the worm motor neurons. Expression of *FUS* wild type did not cause aggregation but expression of mutant *FUS* caused aggregation accompanied by paralysis, neuronal synaptic impairment and neurodegeneration (Vaccaro et al., 2012b).

**Table 4:** **Summary of transgenic FUS models**.

**Study**	**Promoter**	**Gene**	**Motor phenotype**	**Aggregation**	**Neuro-degeneration**	**Synaptic dysfunction**
Murakami et al., 2012	*rgef-1*: entire nervous system	FUS^WT^	No	No	n.d.	n.d.
		FUS^R514G^	No	No	n.d.	n.d.
		FUS^R521G^	No	No	n.d.	n.d.
		FUS^R522G^	Yes	Yes	n.d.	n.d.
		FUS^P525L^	Yes	Yes	n.d.	n.d.
		FUS^501trunc^	Yes	Yes	n.d.	n.d.
		FUS^513trunc^	Yes	Yes	n.d.	n.d.
Vaccaro et al., 2012c	*unc-47*: GABAergic neurons	FUS^WT^	No	No	No	Normal
		FUS^S57Δ^	Yes	Yes	GABAergic neurons	Impaired

*n.d., not determined; trunc, truncation*.

*FUS* is well conserved and the *C. elegans* ortholog is named *fust-1*. In contrast to *tdp-1*, a *fust-1* deletion mutant could not alleviate the toxicity induced by the expression of C′ TDP-43 fragment (Zhang et al., 2012b), suggesting a different role in proteotoxicity. In *Drosophila*, *Cabeza* (*Caz*), the *Drosophila* ortholog of *FUS*, is expressed in motor neurons and a decreased expression of *Caz* causes a motor phenotype and motor neuron degeneration (Wang et al., 2011a; Sasayama et al., 2012). These results suggest a link between the expression and function of FUS, and the specificity of ALS neurodegeneration and we await further investigations of *fust-1* in *C. elegans*.

## Use of *C. elegans* to model C9ORF72 toxicity

A region of chromosome 9 had been linked to ALS for several years (Morita et al., 2006; Vance et al., 2006; van Es et al., 2009; Shatunov et al., 2010) but the gene was only identified in 2011 (DeJesus-Hernandez et al., 2011; Renton et al., 2011) and has since been shown to be a major cause of sporadic and familial ALS (Turner et al., 2013b). The basis of the mutation is a GGGGCC repeat expansion within the first intron of *C9ORF72*. Many questions remain to be answered about the role of *C9ORF72* in the pathogenesis of ALS. It is still not clear whether the GGGGCC repeat expansion results in a loss of function, a gain of function or both, or if the size of the repeat has differential effects on these potential mechanisms. Recent reports have observed decreased expression of *C9ORF72* when the GGGGCC repeat reaches pathogenic length (DeJesus-Hernandez et al., 2011; Ciura et al., 2013; Xi et al., 2013). Whether decreased expression contributes to ALS pathogenesis is unknown since very little is known about the biological role of C9ORF72 other than its sequence similarity to the GDP/GTP exchange factor “Differentially Expressed in Normal and Neoplasia” (DENN) (Zhang et al., 2012a; Levine et al., 2013). DENN proteins are involved in the regulation of Rab-GTPases and endocytosis. Recently, C9ORF72 was shown to be implicated in endosomal trafficking (Farg et al., 2014), confirming its role as a DENN protein. In *C. elegans*, work has been previously done regarding some Rab proteins using deletion mutants and GFP reporters (Sato et al., 2008) to investigate endocytosis (Fares and Grant, 2002). *C. elegans* would be an ideal model to confirm the involvement of *C9ORF72* in this pathway. The *C. elegans* homolog of *C9ORF72* is named *alfa-1* (**AL**S/**F**TD **a**ssociated gene homolog). Our group characterized the loss of function mutant *alfa-1(ok3062)* where we observed that decreased expression of *alfa-1* causes a motility defect, neurodegeneration specifically of the motor neurons and sensitivity to osmotic stress (Therrien et al., 2013). Further characterization still remains to be done but it is interesting that loss of *alfa-1* is linked to neuronal integrity specifically for GABAergic motor neurons in worm.

GGGGCC repeat expansions are found in the first intron of *C9ORF72* and the presence of such long non-coding repeats is suggestive of a toxic gain of function mechanism driving neurodegeneration as seen in many of the trinucleotide repeat expansion diseases. In patients, the repeat was shown to induce abnormal translation (non-ATG translation of the repeat, also called RAN translation) leading to the production of different dipeptides(Ash et al., 2013; Mori et al., 2013b). Also of interest were the presence of RNA foci containing the expanded GGGGCC repeat in patients (DeJesus-Hernandez et al., 2011). It is unknown whether a toxic gain of function is caused by the presence of toxic RNA or the presence of toxic protein, or both. So far, no groups have generated transgenic worms to model this aspect of the toxicity however the expression of the non-coding GGGGCC repeat in *Drosophila* causes neurodegeneration (Xu et al., 2013). *C. elegans* may be useful to model non-coding repeats based on previous efforts studying the expression of non-coding CUG repeats that were toxic to worms (Chen et al., 2007) and recapitulated aspects of RNA foci toxicity (Wang et al., 2011b).

## Stress response and age-dependent neurodegeneration in *C. elegans*

With the identification of TDP-43 in most ALS aggregates and later the identification of mutations affecting *TARDBP* and *FUS* genes, RNA metabolism has become an important area of investigation in ALS research. Under normal conditions, both proteins are mainly observed in the nucleus but the mutant proteins are also found in the cytoplasm. FUS and TDP-43 contain a low-complexity prion-like domain and a RNA binding domain suggesting a role in RNA metabolism (Li et al., 2013b). High throughput RNA-sequencing experiments have been used to identify targets of TDP-43 and FUS in normal or disease states. In worms, the transcriptome of the *tdp-1(ok803)* mutant has been studied (Zhang et al., 2012b) and showed the involvement of TDP-1 in various aspects of development.

Under cellular stress, wild type and mutant TDP-43 and FUS proteins form RNA granules (Bosco et al., 2010; Dormann et al., 2010; Liu-Yesucevitz et al., 2010; Gal et al., 2011; McDonald et al., 2011). These granules are usually formed in order to protect RNA from degradation under stress conditions. In worms, a variety of different RNA granules exist: P granules, P bodies and stress granules. P granules are the most characterized RNA granules in worms and are highly involved in cellular development (Updike and Strome, 2010). However, human proteins found in P bodies and stress granules, such as TIA1, the decapping enzymes and polyA binding proteins, have *C. elegans* ortholog and their role seem conserved regarding RNA granules (Jud et al., 2008; Sun et al., 2011). An active area of research concerns whether mutant TDP-43 and/or FUS proteins interfere with stress granule homeostasis. In a transgenic model of FUS, wild type and mutant FUS were shown to colocalize to stress granules after a heat shock but only the recruitment of mutant FUS to the stress granules caused persistent motility defects in the worms (Murakami et al., 2012). Most work done in *C. elegans* to study stress granules have used thermal stress as an inducer of the granule (Sun et al., 2011). In cells, formation of granules containing FUS is also initiated by other environmental stresses such as osmotic stress (Baron et al., 2013) and oxidative stress (Vance et al., 2013), thus the effect of these other stresses would be interesting to evaluate. Since most of the components of the granules are conserved in worms, *C*. *elegans* could be a powerful system to investigate stress biology in the context of aging, an aspect not easily studied in cellular systems.

Since TDP-43 and FUS are components of stress granules, this has led to the hypothesis that both proteins may be involved in the cellular stress response. The genetic pathways governing cellular stress signaling have been studied to great success in *C. elegans*. The different stress response pathways are highly characterized in worms with the insulin/IGF-1 pathway being a major, conserved signaling axis (Lau and Chalasani, 2014). In worms, the insulin/IGF-1 pathway has a sole insulin/IGF-1 receptor, DAF-2, that acts through the kinases AGE-1, PDK and AKT to phosphorylate the FOXO transcription factor DAF-16, and regulate stress resistance and longevity (Lapierre and Hansen, 2012). The most common stresses applied to worms in laboratory settings include exposure to thermal, oxidative, osmotic or hypoxic stresses (Rodriguez et al., 2013). While each is a damaging stress, they can elicit distinct genetic signaling pathways with diverse outcomes. An open question in the field of late-onset neurodegeneration is whether environmental components exist to account for the range in disease onset and progression for what are many highly penetrant, monogenic, dominantly acting disorders. A stress intrinsic to ALS and many neurodegenerative diseases is proteotoxicity. Here mutant proteins misfold leading to a diverse range of proteotoxic consequences. Thus, cells maintain an extensive network of mechanisms, including the insulin/IGF-1 pathway, to maintain protein homeostasis in the face of environmentally derived damage or genetically encoded misfolded proteins.

Work from *C. elegans* directly linked *tdp-1* to the insulin/IGF-1 pathway and proteotoxicity. In *C. elegans tdp-1* is required for the stress resistance of *daf-2* mutants and the stress-induced expression of *tdp-1* was dependent on *daf-16*. These data suggest a role for TDP-1/TDP-43 in the insulin/IGF-1 pathway and it remains to see if insulin/IGF-1 signaling is altered by disease-associated TDP-43 mutations.

Interestingly, in Vaccaro et al., mutant TDP-43 and mutant FUS proteins were only expressed in the 26 GABAergic motor neurons but activated the ER unfolded protein response chaperone HSP-4 in intestinal tissue (Vaccaro et al., 2012c). This observation suggests that proteotoxic insults can induce stress-signaling pathways in other tissues. It is not known if this is due to a diffusible signaling molecule, or if the mutant proteins make their way from the nervous system to adjacent tissue. TDP-43, FUS, HNRNPA1, HNRNAP2B and TAF15 all contain a prion-like domain (Couthouis et al., 2011; Polymenidou and Cleveland, 2011; Kim et al., 2013) and misfolded SOD1 protein may be able to self propagate (Grad and Cashman, 2014). Thus, these proteins could share properties with toxic prion protein (PrPsc) that misfolds, become infectious, and spreads from cell to cell (Kabir and Safar, 2014). The development of ALS symptoms, starting usually in the lower limb and spreading upward, also suggests a propagation mechanism. Little is known about the propagation potential of the ALS associated misfolded proteins in *C. elegans* transgenic models. Mutant TDP-43 and FUS proteins in the worm's motor neurons were shown to induce the expression of HSP-4 in the intestine, but the proteins were not visualized outside of the neurons where they were expressed (Vaccaro et al., 2012c). A prion model was however characterized expressing Sup35NM, a yeast prion protein, in the body wall muscle of the worm. The most toxic form of the protein was shown between muscle cells, in the intestine and the coelomocytes, and the toxic fibrils were able to induce protein misfolding (Nussbaum-Krammer et al., 2013). Also, proteostasis, ER stress resistance and longevity, all major ALS research topics, have been recently shown to have important cell-non-autonomous components (Taylor and Dillin, 2013; van Oosten-Hawle et al., 2013). Since *C*. *elegans* is transparent, direct visualization of tagged proteins during development and aging is possible. The development of additional tools should help establish if a propagation mechanism exists for mutant TDP-43, FUS and SOD1 proteins.

## Identification of genetic interactions

**Figure 1:**
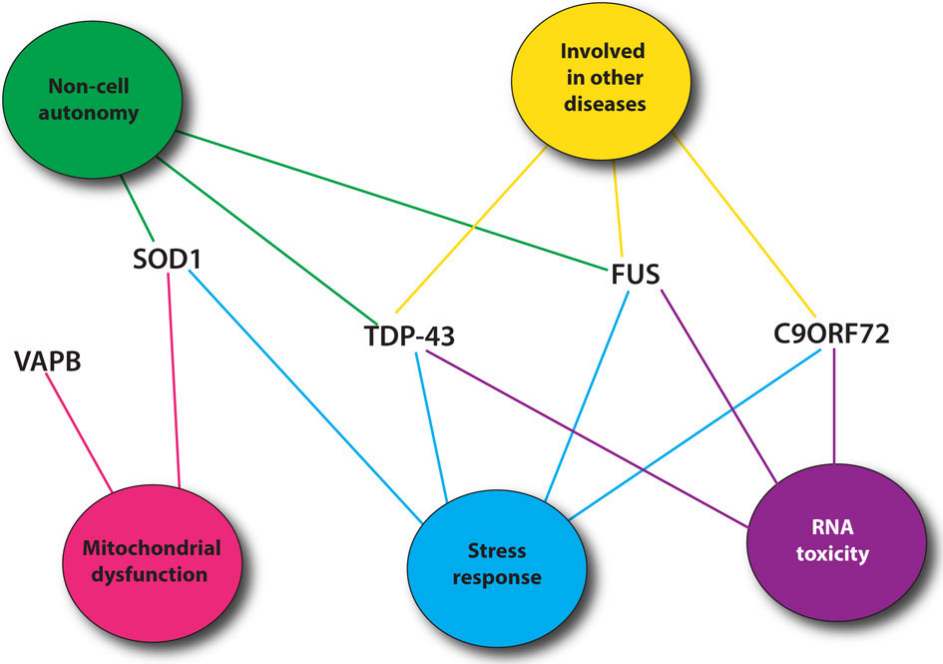
**Most common ALS genes and pathogenic pathways**. Shown are mutant proteins associated with ALS and putative, shared toxicity mechanisms including RNA toxicity, the cellular stress response, mitochondrial impairment and cell autonomy/non-autonomy. Many of these pathways can be easily investigated using *C. elegans*.

Recent genetic advances have identified many new causative genes for familial cases of ALS (Table 1). Moreover, genome-wide association studies (GWAS) have also been done in sporadic ALS cohorts to identify potential genes (Renton et al., 2014). With the increasing number of genes linked to ALS along with the diverse functions of these genes, it is essential to identify common pathological pathways relevant to ALS. Genetic interactions amongst genes can refer to functional relationships amongst a group of genes (Boucher and Jenna, 2013). However, genetic interactions are not always easy to interpret and do not necessarily point toward genes that function in the same pathway but rather identify functional similarity between genes that could be in the same pathway or in compensatory pathways (Boucher and Jenna, 2013). Therefore, identification of genetic interactions between ALS genes could point toward potential therapeutic avenues for ALS patients (Figure 1).

Among the proteins identified, TDP-43, SOD1, FUS, OPTN, UBQLN2, and NEFH proteins are found in familial and sporadic ALS inclusions (Al-Chalabi et al., 2012). In zebrafish and *Drosophila*, FUS, and TDP-43 were shown to interact genetically together but independently of SOD1 (Kabashi et al., 2011; Lanson et al., 2011; Wang et al., 2011a). The rapid development of phenotypes and the availability of multiple mutants or RNAi clones make *C. elegans* an expedient model to study genetic interactions. In worms, TDP-1 was shown to participate in the neurotoxicity observed in motor neuron caused by human TDP-43 and human FUS (Vaccaro et al., 2012c). However, FUS and TDP-43 seem to interact differently with PGRN, a gene involved in FTD, and C9ORF72/ALFA-1 (Tauffenberger et al., 2013a; Therrien et al., 2013). Those results provide an interesting start to the identification of common pathological pathways in ALS. Finally, the characterization of the loss of function mutant of *pgrn-1*, the *C. elegans* ortholog of progranulin, showed that PGRN-1 is involved in apoptotic cell clearance (Kao et al., 2011). Understanding how *pgrn-1* interacts with the different genes involved in ALS/FTD could help to better understand the variation observed along ALS/FTD continuum (Mackenzie et al., 2011).

At a broader level, screening for potential genetic modifiers using RNAi has brought a new understanding of the pathogenesis. For example, SOD1 aggregation was linked to motor dysfunction in worms, but upon decreasing the expression of chaperone proteins, the worms exhibited larger aggregates and increased locomotion deficits (Wang et al., 2009). A non-biased screening approach has recently demonstrated that targeting proteins that affect the phosphorylation levels of TDP-43 greatly affects its neuronal toxicity, setting the stage for novel therapeutic approaches (Liachko et al., 2013). Thus far, only a handful of genetic screens have been described for *C. elegans* ALS models but future studies may help uncover pathogenic mechanisms and therapeutic strategies.

TDP-43 aggregates were shown to be the main protein found in non-SOD1 ALS cases (Neumann et al., 2006). However, the presence of TDP-43 aggregates is not exclusive to ALS. TDP-43 aggregates are observed in other neurodegenerative diseases such as Huntington's disease, Parkinson's disease, Alzheimer's disease, and FTD (Mackenzie et al., 2010). FUS is also found in the aggregates of polyglutamine disorders (Woulfe et al., 2010) and mutations in *FUS* were linked to essential tremor (Merner et al., 2012). Recently, FIG4 and VCP were also identified in different neurodegenerative aggregates (Mori et al., 2013a; Kon et al., 2014). Uncovering specific genetic interactions that involve these proteins could also help our understanding of their recruitment to the aggregates of so many neurodegenerative disorders. Using other models, groups have shown that intermediate polyglutamine repeat of *ATXN2* gene and EPHA4 are potent modulators of ALS toxicity (Elden et al., 2010; Van Hoecke et al., 2012). Therefore, a genetic interaction map may extend the role of these genes beyond ALS and perhaps into other neurodegenerative disorders.

## Using *C. elegans* model for ALS drug discovery

The small size of *C. elegans*, its rapid life cycle, its ease of cultivation and ability to obtain large numbers of animals makes it an attractive model for drug discovery. Furthermore, worms can be grown on solid media or in liquid culture, the latter being relatively easy to adapt for drug screening purposes often through use of multiwell plates and/or automated screening methods (O'Reilly et al., 2013). The transparency of *C. elegans* makes it an ideal model for neurodegeneration applications since protein aggregation and neuronal morphology can be easily assayed as a complement to behavioral phenotypes.

Boyd and colleagues have shown that drugs identified from cell based systems often have relevance in *C. elegans* (Boyd et al., 2013). A screen to identify compounds that decrease TDP-43 aggregation was performed in cell lines and many of the molecules identified were able to suppress the impaired motility phenotype of worms expressing mutant human TDP-43 (Boyd et al., 2013).

Our group has also developed a high-throughput drug-screening assay. We observed that the paralysis phenotype that typically manifests over 5–12 days on solid media can be observed after just hours when the worms are placed in liquid culture (Vaccaro et al., 2012b; Therrien et al., 2013). Using this technique, more than 4000 FDA approved compounds were screened in our laboratory. From this screen, we identified a number of molecules including methylene blue and others acting on the ER stress response that decrease the toxicity of TDP-43 (Vaccaro et al., 2012a, 2013). Interestingly, these drugs were also confirmed in zebrafish ALS models confirming that these compounds can be effective across species. These compounds are therefore promising leads for testing in mammalian models.

Even though in the disease state, aging and neurodegeneration seem to go hand in hand, we have shown that the drugs that act on neurodegeneration can be separated from those that broadly affect lifespan (Tauffenberger et al., 2013b) suggesting that lifespan extension is not a strong predictor of neuroprotection.

## Other motor neuron diseases

ALS is part of the neurological group of disorders called motor neuron diseases. This group also includes spinal muscular atrophy (SMA), primary lateral sclerosis (PLS), hereditary spastic paraplegia (HSP) and many others affecting the upper and/or lower motor neurons. The causative genes of these diseases are involved in many cellular functions, however they all share a common toxic pathways since they mainly affect motor neurons. Finding similarities and differences among those diseases could highly increase our understanding of motor neuron toxicity. *C. elegans* has been used to study two of these, SMA and HSP.

HSP is a group of disorders affecting mainly the lower motor neurons. More than 40 loci have been linked to HSP and the genes identify are involved in axon pathfinding and myelination, mitochondrial maintenance and membrane trafficking (Blackstone, 2012). Recently, a large network including many of these genes have been identify and this network is highly similar to Parkinson's, ALS and Alzheimer's diseases (Novarino et al., 2014). Using *C. elegans*, the function and toxicity of two HSP genes have been investigated. First, *spas-1*, the *C. elegans* ortholog of *spastin*, also called *SPG4*, was shown to be involved in the development of microtubules. SPAS-1 is expressed in the cytoskeleton and is involved specifically in the disassembly of microtubules (Matsushita-Ishiodori et al., 2007). Then, the pan neuronal expression of *NIPA-1* associated mutations led to motor deficits and shortened the lifespan of transgenic worms probably through the activation of caspases and increased ER toxicity (Zhao et al., 2008). With the rapid discovery of new HSP genes, more models are surely to come and will help unravel similarities between these diseases.

SMA is a rare autosomal recessive disorder and a leading genetic cause of infant death. All genetic causes of SMA lead to a decreased expression of the proteins survival of motor neuron (SMN) 1 and 2 (Arnold and Burghes, 2013). It mainly affects the lower motor neurons, but recent evidences suggest that it can be a systemic disease affecting the vascular, cardiac and hepatic functions as well as affecting bone formation (Hamilton and Gillingwater, 2013). *C. elegans* possesses one ortholog of the *SMN* gene, *smn-1*. In 1999, Miguel-Aliaga and colleagues showed that decreased expression of *smn-1* in worms resulted in severe locomotion defects and sterility (Miguel-Aliaga et al., 1999). Then SMN-1 was shown to interact with SMI-1, a known interactor of SMN in humans (Burt et al., 2006). Briese and colleagues characterized the first *smn-1* deletion mutant observing that the mutants displayed early developmental arrest, which could be rescued by reintroducing expression of *smn-1* in the nervous system, while expression in muscle cells was ineffective (Briese et al., 2009). Little is known about any downstream targets of SMN and no drugs are available. Thus, several groups have used *C. elegans* to identify modifiers of the *smn-1* phenotypes. In a cross-species study, it was shown that proteins involved in endocytosis and mRNA regulation could modify the toxicity (Dimitriadi et al., 2010). Also, knowing that the ubiquitin-proteasome pathway degrades SMN, decreased expression of *Mibl*, an E3 ligase, was shown to ameliorate *smn-1* phenotypes (Kwon et al., 2013). Since the *smn-1* deletion allele o*k355* is an early larval lethal phenotype, to aid the development of drug screening Sleigh and colleagues identified a less severe mutant allele that more closely resembles the severity of SMA (Sleigh et al., 2011). Using this mutant, they identified several small molecules that alleviate *smn-1* phenotypes of the worms, therefore, being highly promising compounds for SMA drug development (Sleigh et al., 2011).

## Perspectives

With the discovery of many new ALS genes comes the need to better understand their functions, expression patterns and their modes of toxicity. *C. elegans* has proven to be an informative model to study neurodegeneration mechanisms arising from multiple ALS related proteins. We envision that the introduction of new transgenic and genetic models will help unravel important questions about the normal and pathogenic roles of these proteins.

Most models explained here recapitulate some if not many, important features of ALS, however, phenotypic variations are seen amongst the different models, for a number of reasons. First, the models do not all use the same mutations, thus the resulting mutant proteins may not all be equally toxic, or display the same interactions with other proteins. Also, the level of expression is important to consider as for example, there is considerable evidence that TDP-43 levels are tightly regulated (Budini and Buratti, 2011), and elevated expression is toxic in nearly every system studied (Ash et al., 2010; Xu et al., 2010; Estes et al., 2011). The most common method to generate transgenic worms is by microinjection to create stable lines followed by radiation to integrate the transgene in the genome. This procedure typically produces transgenics with multiple copies of the gene inserted in the genome, thus some of the toxicity observed may be due to overexpression. Aware of this issue, a new generation of ALS transgenic worms should be constructed based on single copy integration (Frøkjaer-Jensen et al., 2008) or with the CRISPR-Cas9 method (Friedland et al., 2013) instead to ensure that transgenic lines have a similar level of expression from the same genomic location. Finally, the phenotypic variance may also be due to the promoter used. Some models have used pan neuronal expression constructs, while others have targeted transgenic expression to specific neuronal populations. In humans, most of these proteins are expressed ubiquitously but only specific neuronal populations are sensitive to degeneration. Thus, worm models based on motor neuron transgenics could be ideal model to uncover conserved mechanisms of motor neuron degeneration. To confirm the specificity of each phenotype, mutant and wild type proteins should be carefully compared and similar changes should be confirmed in higher eukaryotes. For example, mutant TDP-43 and FUS proteins induce an ER stress response in worms which is not observed when the wild type proteins are expressed (Vaccaro et al., 2012b). Also, the ER stress response was shown to be activated in other ALS models and in patients (see section above).

These models are setting the stage for novel toxicity hypothesis. The immune system seems to play an important role in the neurodegeneration observed in ALS. Protein aggregation could activate the immune response and neuroinflammation actively contributes to disease progression (McCombe and Henderson, 2011). *C. elegans* relies on an evolutionary conserved, innate immune response (Engelmann and Pujol, 2010) that coordinates its activity with the insulin/IGF-1 pathway (Singh and Aballay, 2009) suggesting these may be pathways worth investigating. Also, in the past year, a convergence of data has suggested a role for glial cells in ALS neurodegeneration (Parisi et al., 2013; Valori et al., 2013; Chiu et al., 2014). The worm has 56 glial cells and some are found at the neuromuscular junction (Oikonomou and Shaham, 2011). Characterization of the cross talk between the neurons and the glial cells would also be an interesting area of investigation.

An important topic related to ALS and to other neurodegenerative disorders is aging. The risk of ALS increases with age, peaking between 70 and 80 years old (Gordon, 2013). Aging pathways are well characterized in worms and among others, include the insulin/IGF-1, the target of rapamycin (TOR) and germline signaling pathways. There is a strong overlap between protetotoxicity and aging where autophagy and lipid metabolism are major targets (Lapierre and Hansen, 2012). Evaluating the toxic impact of mutant proteins during aging is not feasible in many models, but is easily accomplished using *C. elegans*. The development of models with age-related toxicity is essential and could help understand the link between the proteotoxicity and aging.

When using *C. elegans* or other animal models, most studies have focused on the toxicity of known ALS genes. It is important to note that almost 90% of ALS cases are sporadic ALS with no link to known genetic abnormalities. Therefore, we still do not know how most patients develop ALS. However, it is important to know that sporadic and familial cases of ALS are clinically indistinguishable (Al-Chalabi and Hardiman, 2013). Given that ALS patients can live between 6 months and 6 years after diagnosis, it has been hypothesized that environmental factors may influence disease onset and progression (Al-Chalabi and Hardiman, 2013). Many environmental factors have been examined in relation to ALS but there is no consensus for their contribution to the disease (Al-Chalabi and Hardiman, 2013). *C. elegans* could be useful to study some of the environmental risks hypothesized. In fact, several groups have identified compounds that could cause specific degeneration of motor neurons (Du and Wang, 2009; Negga et al., 2012; Estevez et al., 2014) opening the door to identifying environmental modifiers of degeneration in ALS models.

However, how relevant are any of these findings to humans? Will any of the drugs identified in *C. elegans* translate to mammalian models let alone ALS patients? So far, many drugs identified using rodent models focusing mainly on protein aggregation and cell death mechanisms have failed in subsequent clinical trials. Using *C. elegans* to identify drugs acting on early neuronal dysfunction mechanisms could be an effective way to prevent ensuing cellular decline and death. From a liquid culture screen, our group has identified a compound with this property (unpublished results). The compound is effective in vertebrate ALS animal models and is now being tested in ALS patients. Therefore, large screens using *C. elegans* targeting specific early aspects of neurodegeneration seem promising and show relevance in higher organisms.

### Conflict of interest statement

The authors declare that the research was conducted in the absence of any commercial or financial relationships that could be construed as a potential conflict of interest.
